# Effects of the Manufacturing Methods on the Mechanical Properties of a Medical-Grade Copolymer Poly(L-lactide-co-D,L-lactide) and Poly(L-lactide-co-ε-caprolactone) Blend

**DOI:** 10.3390/ma14216381

**Published:** 2021-10-25

**Authors:** Mariana Rodriguez Reinoso, Marco Civera, Vito Burgio, Annalisa Chiappone, Oliver Grimaldo Ruiz, Alessandra D’Anna, Carmela Riccio, Ignazio Roppolo, Alberto Frache, Paola Antonaci, Cecilia Surace

**Affiliations:** 1 Laboratory of Bio-Inspired Nanomechanics, Department of Structural, Geotechnical and Building Engineering, Politecnico di Torino, 10129 Turin, Italy; mariana.rodriguez@polito.it (M.R.R.); s255602@studenti.polito.it (V.B.); oliver.grimaldo@polito.it (O.G.R.); s251391@studenti.polito.it (C.R.); paola.antonaci@polito.it (P.A.); cecilia.surace@polito.it (C.S.); 2 Department of Mechanical and Aerospace Engineering, Politecnico di Torino, 10129 Turin, Italy; 3 Department of Applied Science and Technology, Politecnico di Torino, 10129 Turin, Italy; annalisa.chiappone@polito.it (A.C.); alessandra.danna@polito.it (A.D.); ignazio.roppolo@polito.it (I.R.); alberto.frache@polito.it (A.F.)

**Keywords:** biodegradable, polymer, lactide, caprolactone, injection and compression molding, mechanical properties

## Abstract

Biocompatible and biodegradable polymers represent the future in the manufacturing of medical implantable solutions. As of today, these are generally manufactured with metallic components which cannot be naturally absorbed within the human body. This requires performing an additional surgical procedure to remove the remnants after complete rehabilitation or to leave the devices in situ indefinitely. Nevertheless, the biomaterials used for this purpose must satisfy well-defined mechanical requirements. These are difficult to ascertain at the design phase since they depend not only on their physicochemical properties but also on the specific manufacturing methods used for the target application. Therefore, this research was focused on establishing the effects of the manufacturing methods on both the mechanical properties and the thermal behavior of a medical-grade copolymer blend. Specifically, Injection and Compression Molding were considered. A Poly(L-lactide-co-D,L-lactide)/Poly(L-lactide-co-ε-caprolactone) blend was considered for this investigation, with a ratio of 50/50 (*w/w*), aimed at the manufacturing of implantable devices for tendon repair. Interesting results were obtained.

## 1. Introduction

In the last few years, medical-grade copolymers have been used in an increasingly large number of biomedical applications, including tissue engineering scaffolds, implantable medical device manufacturing, textile-based medical solutions, and drug delivery platforms [1,2,3,4,5]. These types of biomaterials, known as biodegradable polymers, enable several advantages, such as controlled degradation rates, precise strength, elasticity, and other mechanical and chemical properties. In particular, Lactide and Caprolactone polymer families are arguably the most widely used among biodegradable materials in the biopharma and medical industry [6,7,8]. These functional polyesters and their tailored blends at different ratios undergo completely natural biodegradation by hydrolysis without releasing harmful compounds to the human body under physiological conditions. Furthermore, biodegradable polymers enable the gradual recovery of tissue function as the material is degraded, as a result a second surgery to remove them can be avoided, reducing medical costs and patient discomfort [9,10,11]. These materials are unique in this compared to commonly used traditional materials, such as metals and ceramics. In addition, biodegradable copolymers can be easily processed in any biomedical application through various transformative methods of additive and subtractive manufacturing (AM-SM), thus allowing the building of complex geometric shapes. However, depending on the target application, the most suitable manufacturing approach along with material selection and optimization must be considered. These parameters will lead to the application effectively ensuring its purpose in vivo and being removed hydrolytically from the native biological system in the projected time [12]. As regards the selection and optimization of the chosen material, although significant results have been obtained with the use of synthetic biodegradable polyesters in biomedical applications, the development of novel biodegradable polymer blends that possess certain chemical-mechanical characteristics is still challenging. In this context, melt blending of polymers is the most simple and cost-effective method aimed to enlarge the spectrum of biodegradable polyesters for the production of biodegradable and biocompatible implantable medical devices. Certainly, polymer blends exhibit functional properties, improved characteristics, and performance that their separate components do not exhibit when used as single materials [13,14]. Lastly, the final properties of the polymer blend will depend on several factors, such as blending ratio, the presence of additives/compatibilizers, and melt blending processing parameters (e.g., temperature and pressure).

This research focused on establishing the effects of two manufacturing methods, Compression Molding (CM) and Injection Molding (IM), on the mechanical properties of a biodegradable polyesters blend. These two methods represent the most used manufacturing processes in the polymer industry. Specifically, it has been investigated in a 50/50 (*w*/*w*) ratio for the manufacturing of implantable devices for tendon repair. Consequently, two tailored synthetic copolymers, Poly(L-lactide-co-D,L-lactide) (P(L/DL)LA) and Poly(L-lactide-co-ε-caprolactone) (PLCL), were strategically selected to match the elastic modulus of the human Achilles tendon (300–1400 MPa) [15]. P(L/DL)LA has been chosen to achieve favorable mechanical properties for medical implantable devices manufacturing, with a modulus of elasticity ranging between 1900 and 2400 MPa and maximum yield stress of about 35 MPa. The amorphous structure of P(L/DL)LA, arising from the random distribution of the lactide monomers, was also expected to contribute positively to the elongation properties [16]. It has been considered adding to the blend a more ductile material with a lower modulus of elasticity such as PLCL, which could be tailored to the target elastic modulus of the medical device material. In particular, the values of the elastic modulus are as much lower as the PCL content is higher. Studies performed on the mechanical properties of this material report a modulus of elasticity of about 239–352 MPa and yield stress of 12–16 MPa were observed [17,18,19]. The P(L/DL)LA/PLCL blend was obtained by using a twin-screw extruder, due to its high efficiency, its short manufacturing time, and high productivity [20]. Subsequently, CM and IM were employed.

Mechanical, thermal, and morphological characterization was performed as well, using the uniaxial tensile test, differential scanning calorimetry (DSC), and scanning electron microscopy (SEM) to assess the specimens.

## 2. Materials and Methods

### 2.1. Medical-Grade Copolymers

The block biodegradable medical-grade copolymers used in this study were supplied in pellets by *Evonik* Industries (Essen; www.evonik.com). Polymer main properties declared by the manufacturer are reported below: Resomer® LR 704 S, Poly(L-lactide-co-D,L-lactide) 70:30. Amorphous material, Inherent viscosity (Iv) 2.0–2.8 dL/g, glass-transition temperature (Tg) 56–62 °C, melting point (mp) 102–111 °C. Resomer^®^ LC 703 S, Poly(L-lactide-co-ε-caprolactone) 70:30. Semi-crystalline material, Inherent viscosity (Iv) 1.3–1.8 dL/g, glass-transition temperature (Tg) 32–42 °C, melting point, (mp)–162–169 °C.

### 2.2. P(L/DL)LA/PLCL Blend Preparation

The preparation of the polymer blend involved several steps, including vacuum drying, melt blending, and final grinding. The pellets of both P(L/DL)LA and PLCL in a 50/50 (*w/w*) ratio were initially dried overnight at 70 °C (<mp) using a vacuum drying ISCO NSV 9090™ oven. After the vacuum drying thermal process, the polymeric blend was prepared by melt extrusion using a Thermo Scientific™ Pharma 11 Twin-screw extruder. An extruder nozzle outlet pressure at 40 bar and a flow rate fixed at 200 g/h were set. The barrel temperature was set from 150 °C to 190 °C along the extruder axis and the melt temperature was measured to be 183 °C. At the end of the extrusion process, the PLA/PCL blend leaving the extruder was passed in a cooling tank and pelletized.

### 2.3. Compression and Injection Molding

Dog-bone specimens Type 5A (specimen design according to ISO 527-2 standard) for each manufacturing method were produced, aimed at mechanical, thermal, and morphological characterizations. The specimens by CM were prepared using a step mold closure at 100 bar, 180 °C for 4 min using a laboratory press (P 200 T COLLIN ™). The specimens by IM were prepared using a Micro-Injection Molding BABYPLAST 610P Horizontal™ machine with a melt temperature of 180 °C, injection time of 10 s at 90 bar, and 20 s at 70 bar; the mold temperature was set at 18 °C.

### 2.4. Differential Scanning Calorimetry of the Copolymers, and P(L/DL)LA/PLCL Blend

The differential scanning calorimetry (DSC) thermal tests were performed on the base materials P(L/DL)LA and PLCL as well as the P(L/DL)A/PLCL with a ratio (*w/w*) of 50/50 in the form of pellets and manufactured by compression and injection molding. The thermal behavior was observed using a Mettler Toledo DSC1 Star System®, the samples (10–20 mg) were inserted into aluminum pans, and scans were performed between 0 °C and 200 °C (first heating; cooling; second heating) at a heating rate of 10 °C/min under nitrogen atmosphere. According to the convention, exothermic phenomena are represented as upward peaks.

### 2.5. Mechanical Characterization of the P(L/DL)LA/PLCL Blend by Uniaxial Tensile Test

**Figure 1:**
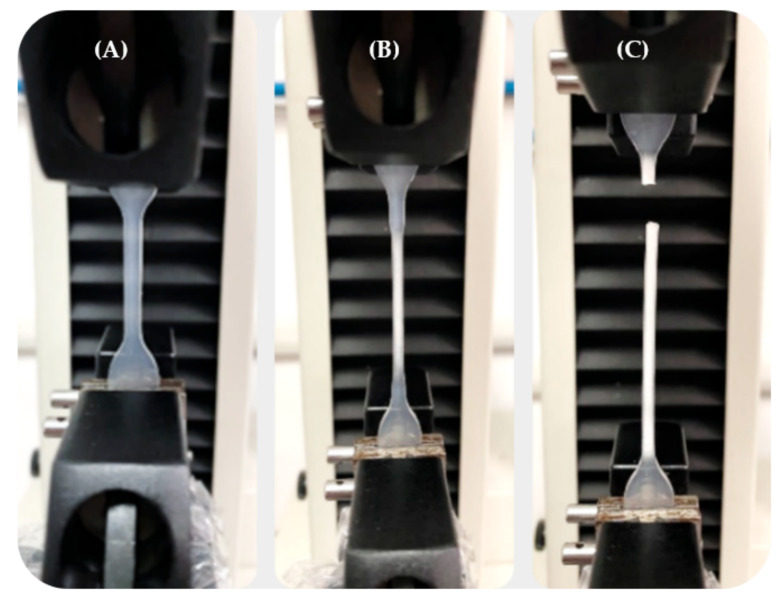
Tensile test on a P(L/DL)LA/PLCL specimen obtained by IM: (**A**) Specimen in the elastic deformation stage; (**B**) Specimen in the plastic deformation stage; (**C**) Specimen at failure.

The mechanical characterization of the P(L/DL)LA/PLCL blend was conducted following the uniaxial tensile testing method described in the Standard ISO 527-1 for plastics. The machine used for the tests was an *MTS* Insight® Electromechanical Testing Systems. For this study, a 1000 N load cell was used. The dog-bone specimens Type 5A required by the ISO 527-2 standard for each manufacturing method (CM and IM) were subjected to uniaxial tensile stress along their longitudinal axis. A distance between grips of 30 mm, sampling frequency of 30 Hz, and constant speed of 0.3 mm/min were set until specimen failure. During the tests, force and displacement values were recorded using the TestWorks^®^ Software-MTS Systems Corporation. Figure 1 shows a tensile test for a dog-bone specimen obtained by IM. It shows the elongation of the specimen in the initial elastic deformation stage, intermediate plastic deformation stage, and failure.

### 2.6. Scanning Electron Microscopy Characterization of the P(L/DL) LA/PLCL Blend

Morphology at the micrometric level is a key factor to understand the macroscopic properties of the blend. CM and IM surfaces obtained through fracturing in liquid nitrogen and coated with a thin platinum layer were examined by *Zeiss-Supra 40 VP/Gemini Column* scanning electron microscope (SEM) using an acceleration voltage of 5 kV.

### 2.7. X-ray Powder Diffraction (XRD)

X-ray diffraction was used to compare the crystalline structure of P(L/DL)LA/PLCL and the blend. To provide information about the changes in the crystalline structure of the polymer after mixing, particularly, the effects of mixing of the two polymers at a molecular level, X-ray diffraction-analyses (XRD) were performed on specimens using PANalytical X’PERT PRO with Cu–KX-ray source (1.540562 Å) and a scanning rate of 0.026°min^−1^.

## 3. Results

The stress, strain, and tensile modulus of elasticity were calculated as described in the ISO 527-1 standard. Table 1 reports the average values of modulus of elasticity Et, stress and strain at yield (σy–εy), stress and strain at break (σb–εb), and their associated standard deviation. Besides, the results of the thermal and morphological tests performed on the copolymers and the blending manufactured by CM and IM are presented.

### 3.1. Tensile Mechanical Properties of the P(L/DL)LA/PLCL Blend Produced by Compression and Injection Molding

**Figure 2:**
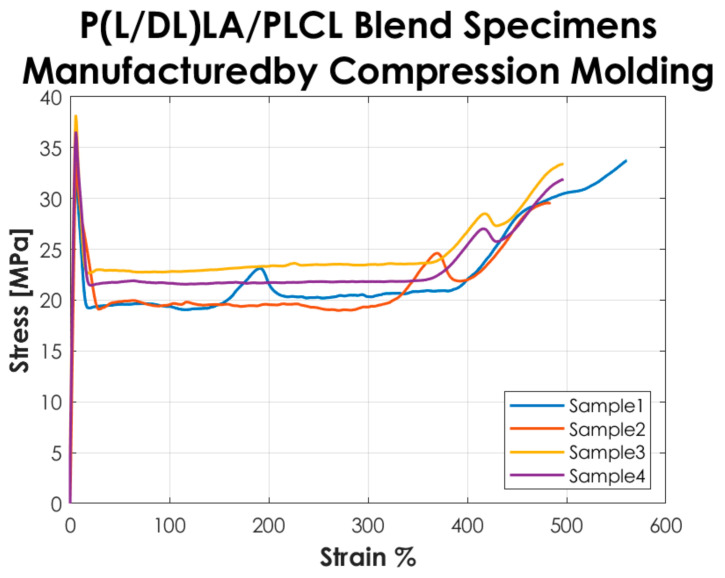
Stress-strain curves resulting from the tensile tests on the specimens manufactured by CM.

Figure 2 shows the stress-strain curves resulting from the tensile tests performed according to the ISO 527-1 standard on the Type 5A specimens manufactured by CM. The curves represented in the graph show a “Type 2” mechanical behavior as defined in the standard, i.e., a strain-hardening behavior, with yield point followed by stress increase.

**Figure 3:**
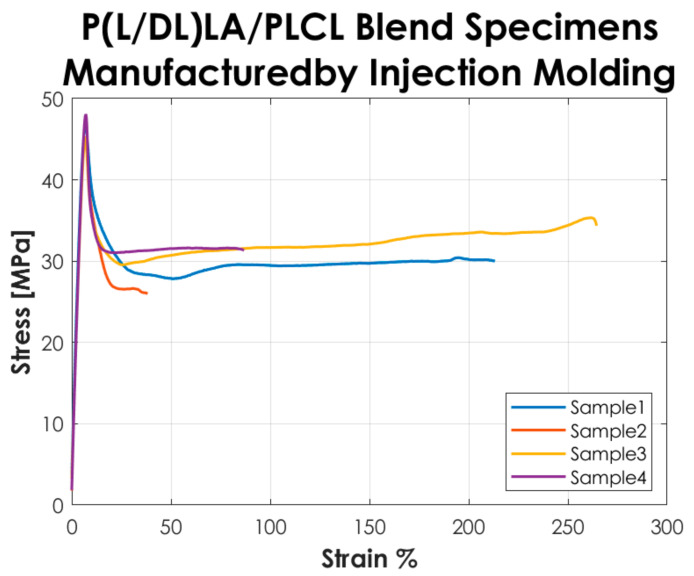
Stress-strain curves resulting from the tensile tests on the specimens manufactured by IM.

Figure 3 shows the stress-strain curves resulting from the tensile tests performed according to the ISO 527-1 standard on the Type 5A specimens manufactured by IM. The curves represented in the graph show a “Type 3” mechanical behavior as described in the standard, i.e., well-defined yield point without stress increase after yielding.

### 3.2. Differential Scanning Calorimetry of the Copolymers and P(L/DL)LA/PLCL Blend

**Figure 4:**
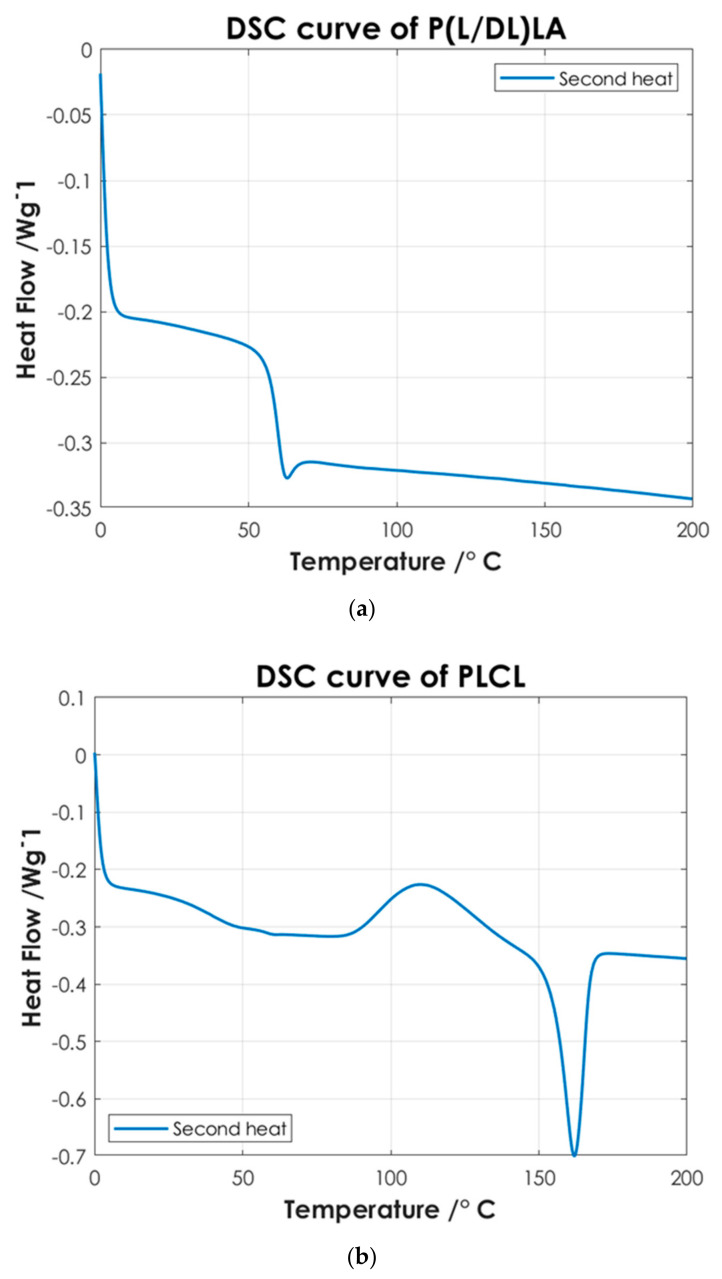

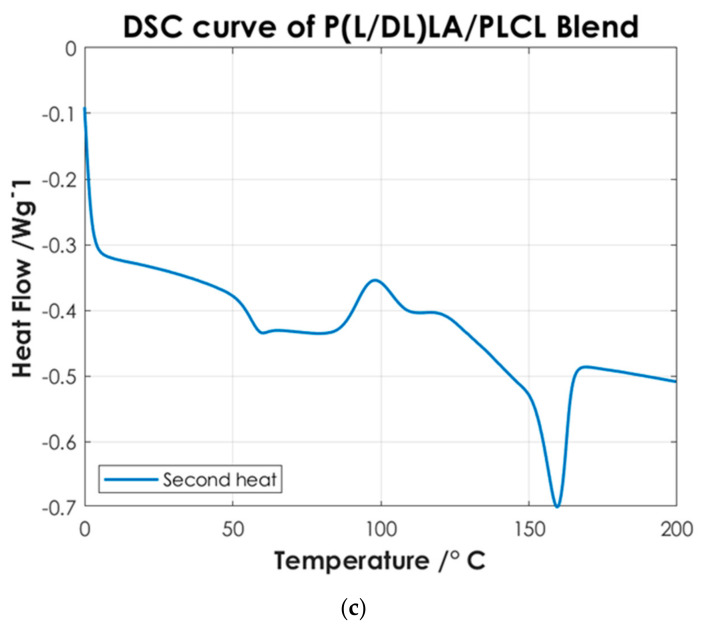
DSC curves of: (**a**) P(L/DL)LA; (**b**) PLCL; and (**c**) P(L/DL)LA/PLCL blend.

**Figure 5:**
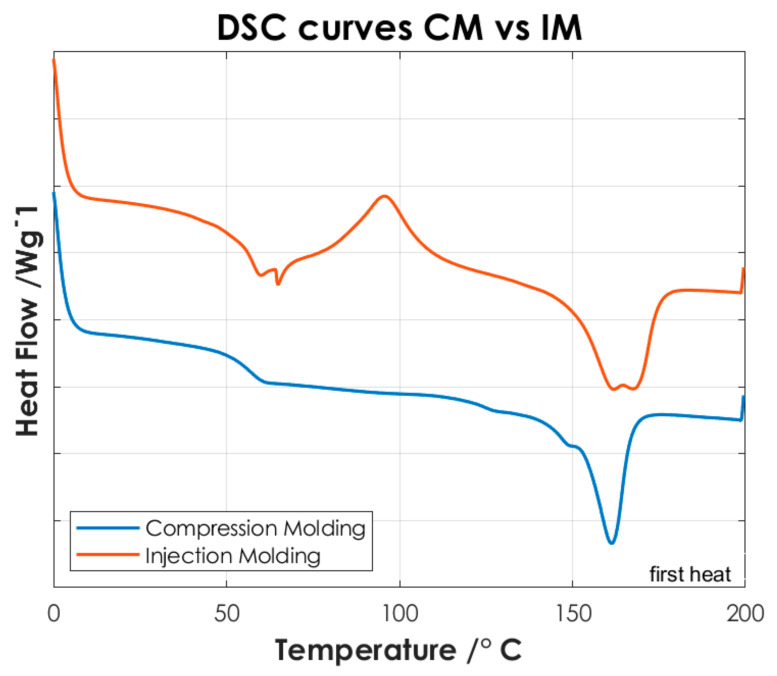
DSC curves of P(L/DL)LA/PLCL blend manufactured by CM and IM (Specimens). For better visualization, an offset of −0.2 has been applied to the data relative to the CM.

To identify the temperatures associated with the characteristic thermal phase transitions of the copolymers and P(L/DL)LA/PLCL blend, the second DSC heating curves were analyzed. Once the samples are subjected to a cooling and subsequent heating cycle (second heating), the intrinsic thermal properties of the materials are largely restored, Figure 4 shows the thermal test results. Finally, the DSC analysis of the manufactured specimens by CM and IM was conducted evaluating the first DSC heating curves. Figure 5 shows the differences between the two manufacturing methods.

### 3.3. Scanning Electron Microscopy Characterization of the P(L/DL)LA/PLCL Blend

**Figure 6:**
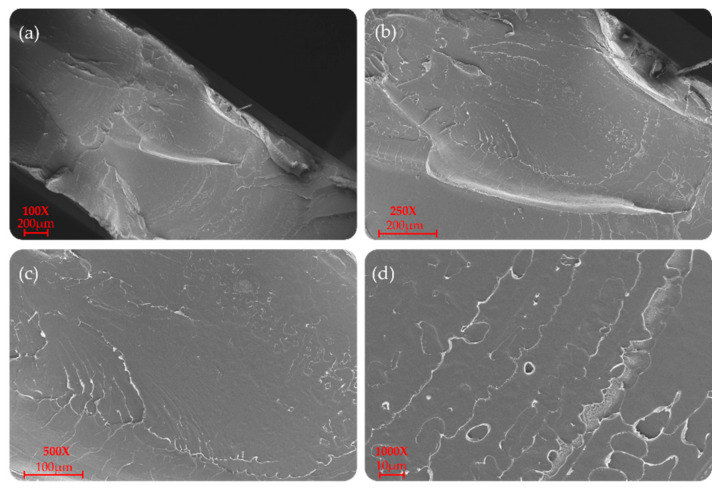
SEM morphology of the surfaces of the P(L/DL)LA/PLCL blend: (**a**–**d**) images correspond to specimens manufactured by CM at different magnifications.

**Figure 7:**
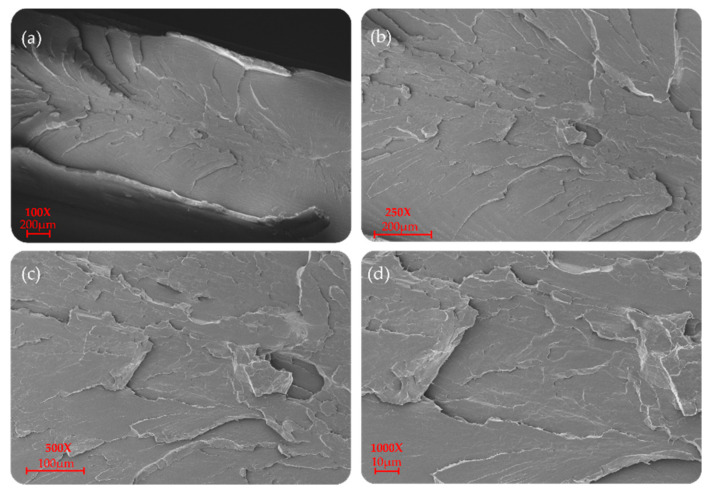
SEM morphology of the surfaces of the P(L/DL)LA/PLCL blend: (**a**–**d**) images correspond to specimens manufactured by IM at different magnifications.

P(L/DL)LA/PLCL blend manufactured by CM and IM is shown at different magnifications (100×, 250×, 500×, and 1000×) in Figure 6 and Figure 7. In particular, a uniform and unique phase of the P(L/DL) LA/PLCL blend can be seen in both cases. No evident materials separation between blend components could be observed, suggesting the physical compatibility of copolymers in the blend.

### 3.4. X-ray Powder Diffraction (XRD)

**Figure 8:**
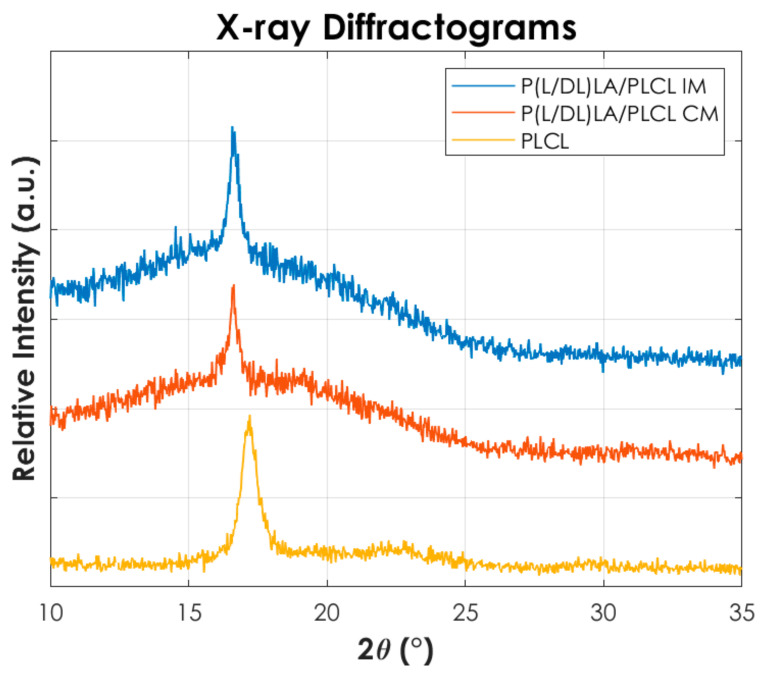
X-ray diffraction-analyses (XRD) for PLCL and the blend manufactured by Injection molding and Compression molding.

Figure 8 shows the X-ray diffraction pattern of the PLCL and the samples of the blend P(L/DL)LA/PLCL obtained by CM and IM. P(L/DL)LA is not reported since it has an amorphous structure.

## 4. Discussion

The tensile test results reported in Figure 2 and Figure 3 showed that the tested specimens had a different mechanical behavior after the yielding point. In particular, compression-molded specimens evidenced a typical trend of ductile strain-hardening materials since there was a plastic zone where a slow increase of stress above the yield value was manifested. On the other hand, injection-molded specimens showed a fast strain-softening after the yielding point, followed by a limited increase of deformation at almost constant load up to failure. Analyzing in detail the values reported in Table 1, it is possible to highlight the following considerations on the results obtained.

In general, the specimens obtained by CM showed a lower modulus of elasticity, stress, and strain at yield compared to those obtained by IM. In particular, a decrease of approximately 20% is evidenced for the mentioned parameters. Likewise, analyzing the stress at break, the values for both manufacturing methods are comparable. The values of the deformation at break exhibited a considerable increase (approx. 3 times higher) in CM specimens by comparison to IM specimens. Despite that, analyzing the standard deviation on the deformation at break, it is evidenced that values for both methods are quite high, especially for IM. Calculating the coefficient of variation (CV), a value of 75% was found for the deformation at break of the IM specimens set, which shows a great variability of the results and consequently a low statistical value. This is because two out of four specimens obtained by IM had deformation at break below 100% and the other two over 200%, so on average, the deformation at break resulted much lower than for those obtained by CM, and the coefficient of variation assumed a considerable value. Nevertheless, the major difference in the mechanical behavior as a function of the manufacturing method was manifested in the post-yield region, the CM method seems to be responsible for a strain-hardening behavior, while a strain-softening behavior was manifested by the specimens produced with the IM. All the results presented here did not account for the potential presence of manufacturing defects. Special care was taken to avoid the occurrence of any defect in the production of the specimens. The effects of manufacturing defects in CM and IM will be the subject of future research.

Figure 4 shows the thermoanalytical results of the base materials in the form of pellets. From Figure 4a it is possible to identify P(L/DL)LA characteristic transitions. P(L/DL)LA exhibits a typical glass transition temperature (Tg) of approximately 60 °C, while melting and crystallization temperatures were not detectable, the value of Tg found matches with the datasheet reported by the manufacturer and similar studies reported in the literature [21]. Thus, the typical behavior of the P(L/DL)LA copolymer, which is mainly amorphous, was confirmed. On the other hand, from the DSC curve of the PLCL (Figure 4b) it is possible to identify a glass transition temperature (Tg) of about 60 °C, a crystallization temperature (Tc) of about 110 °C, and a melting peak (Tm) at 160 °C. The temperatures identified are very close to those reported for the PLLA material, since P(L/DL)LA is a 70:30 copolymer, 70% PLLA, and 30% PCL. These values resulting from the DSC are consistent with those reported by the manufacturer and in literature [5,18,22].

Afterwards, the thermal results of the P(L/DL)LA/PLCL blend were analyzed in the form of pellets before the IM and CM. Indeed, second heating was considered to identify the intrinsic properties of the materials after polymer blending. As of Figure 4c, it is possible to identify a glass transition temperature (Tg) of approximately 60 °C, a crystallization temperature (Tc) of about 90 °C, and a melting temperature (Mp) of 160 °C. The presence of a single glass transition (Tg), crystallization (Tc), and melting (Mp) temperatures evidence the presence of a single phase, which confirms that the melt blending was performed efficiently. As mentioned, the DSC analyses of the manufactured materials for CM and IM were conducted to evaluate the effect of manufacturing methods on the thermal properties of the materials as well as to identify possible differences between the two techniques. Figure 5 shows the DSC curves relating to the first heating as this faithfully reproduces the thermal characteristics of the obtained specimens. In both DSC curves, a glass transition temperature (Tg) of approximately 60 °C and a melting point (Mp) at 160 °C are identified, while the crystallization peak (Tc) at about 90 °C can only be identified in the specimen manufactured by IM. From the summary results reported in Table 2, it is possible to state that there is a difference in the presence of crystallinity (Tc), and orientation in the chains of polymers obtained by the two manufacturing methods. Indeed, the IM method enables the orientation of the polymer chains in a preferential direction, favoring the crystallization peak of the material and leading to an improvement of the mechanical properties; in particular, modulus of elasticity, and stress/strain at yield. Likewise, analyzing the specimens manufactured by IM exhibits a smaller strain. It should be highlighted that the strain effects after yielding are ascribed to the presence of an amorphous PLCL fraction in the blended polymer material [23]. During the tensile test, the polymer chains in the amorphous zone are stretched, thus causing an increment at yielding as shown in Figure 3. In contrast, during specimens manufacturing by CM, it is foreseen that the polymer pellets are arranged randomly on the heating plates without considering a preferential direction. As a result, the crystallinity of the blend is reduced. Indeed, the absence of a Tc in the DSC curve (Figure 5) and the lower values of the mechanical properties (Table 2) show that the specimens manufactured by CM present a lesser crystallinity and they cannot preserve the thermal properties of the material. Other studies confirm the results obtained from the tensile tests performed in this research work, as they state that injection-molded specimens enable reaching higher values of yield stress and tensile modulus than compression-molded specimens [24,25]. These differences highlight the strong impact of the production processes on the mechanical properties of the final product, despite quite similar procedural steps followed in both processes, namely: polymer melting, preparation, pressing, and mold cooling.

As for the morphological analyses made to detect a phase difference, the P(L/DL)LA/PLCL blends analyzed at different magnifications with a fixed work distance showed a homogeneous surface morphology, both for CM and IM methods as reported in Figure 6 and Figure 7. In general, both the specimens manufactured by IM and CM present miscibility between the two polymeric phases which allows obtaining a homogeneous morphological structure.

From Figure 8 it is possible to identify that a PLCL homopolymer shows a peak at 2θ value of 17°, in agreement with the peak reported in the literature [26] and associable with the (110) of an orthorhombic unit cell. Conversely, P(L/DL)LA shows an amorphous structure (not reported here) and no peaks are recorded by confirming the typical amorphous behaviour of the copolymer. In contrast, mixtures obtained by both compression and injection moulding show a peak at 2θ values of 16.57°. Furthermore, it can be seen that the peak is more intense in the injection-moulded blend than the compression-moulded one. This finding suggests a higher crystallinity in the injection-moulded samples, consistent with the results of the DSC thermal analysis. In fact, during the injection moulding process, the polymer chains of the blend can orientate themselves more following the flow of the melt, in contrast to the compression moulding process being more difficult to direct the melt flow.

## 5. Conclusions

This study aimed to evaluate two different manufacturing methods with both processes involving the use of heat and pressure, aimed to obtain parts of biodegradable medical-grade blended polymers for tendon repair applications. Specimens Type 5A were manufactured following ISO 527-1 standard with a P(L/DL)LA/PLCL blend with a ratio of 50/50 (*w*/*w*). The results of tensile tests showed a difference in the mechanical behavior between the compression and injection-molded specimens. The results showed a strain-hardening behavior for compression-molded specimens; thus, there is a stress increase before the specimen fails. This is not visible for injection-molded specimens, which showed a strain-softening behavior. On the other hand, the compression-molded specimens showed values of elastic modulus, yield stress, and yield strain lower than those found in the injection-molded specimens.

The differential scanning calorimetry analysis showed a glass transition temperature at about 60 °C and a melting point at 160 °C for both manufactured specimens, while the crystallization peak at about 90 °C was only identified in the IM specimens. Indeed, the IM method enables the orientation of the polymer chains in a preferential direction, favoring the crystallization peak of the material and leading to an improvement of the mechanical properties.

The morphological analysis showed that the specimens manufactured by IM and CM did not evidence phase separation exhibiting a single homogeneous phase. To summarize, the comparison between the two manufacturing methods showed the influence of the different manufacturing techniques on the mechanical behavior of the P(L/DL)LA/PLCL blend and its properties. Hence, the choice of the manufacturing method depends on the desired mechanical properties and ultimately on the specific application for which the blend is intended.

## Figures and Tables

**Table 1:** Average values and standard deviations of the results for the tensile tests on specimens manufactured by Compression Molding (CM) and Injection Molding (IM):( $\bar{X}$ indicates the average value, STD the standard deviation).

	E_t_ [MPa]		σ_y_ [MPa]		Ɛ_y_ %		σ_b_ [MPa]		Ɛ_b_ %	
	$\bar{X}$	**STD**	$\bar{X}$	**STD**	$\bar{X}$	**STD**	$\bar{X}$	**STD**	$\bar{X}$	**STD**
CM	691.10	24.89	35.83	2.17	5.59	0.003	32.27	1.89	503.70	37.59
IM	881.50	10.11	46.34	1.39	6.75	0.25	31.14	2.53	145.45	109.54

**Table 2:** Approximative phase transition temperatures resulting from DSC test of the materials: Tg is the Glass transition temperature; Tc is the crystallization temperature; Mp is the Melting point.

Materials	∼Tg [°C]	∼Tc [°C]	∼Mp [°C]
P(L/DL)LA (Pellet)	60	-	-
PLCL (Pellet)	40	110	160
P(L/DL)LA/PLCL blend (Pellet)	60	90	160
P(L/DL)LA/PLCL blend manufactured by CM	60	-	160
P(L/DL)LA/PLCL blend manufactured by IM	60	90	160–170

## Data Availability

The data presented in this study are available on reasonable request from the authors.
